# Health Measurement Model—Bringing a Life Course Perspective to Health Measurement: The PRISM Model

**DOI:** 10.3389/fped.2021.605932

**Published:** 2021-06-10

**Authors:** Steven Hirschfeld, Elizabeth Goodman, Shari Barkin, Elaine Faustman, Neal Halfon, Anne W. Riley

**Affiliations:** ^1^Department of Pediatrics, Uniformed Services University of the Health Sciences, Bethesda, MD, United States; ^2^Department of Pediatrics, Massachusetts General Hospital, Boston, MA, United States; ^3^Department of Pediatrics, Vanderbilt University Medical Center, Nashville, TN, United States; ^4^Department of Environmental and Occupational Health Sciences, University of Washington, Seattle, WA, United States; ^5^Department of Health Policy and Management, University of California, Los Angeles, Los Angeles, CA, United States; ^6^Department of Population, Family, and Reproductive Health, Johns Hopkins University, Baltimore, MD, United States

**Keywords:** life course health development, health measurement, childhood, measurement models, longitudinal study

## Abstract

Health is a multidimensional concept that is challenging to measure, and in the rapidly evolving developmental changes that occur during the first 21 years of human life, requires a dynamic approach to accurately capture the transitions, and overall arc of a complex process of internal and external interactions. We propose an approach that integrates a lifecourse framework with a layered series of assessments, each layer using a many to many mapping, to converge on four fundamental dimensions of health measurement-Potential, Adaptability, Performance, and Experience. The four dimensions can conceptually be mapped onto a plane with each edge of the resulting quadrilateral corresponding to one dimension and each dimensions assessment calibrated against a theoretical ideal. As the plane evolves over time, the sequential measurements will form a volume. We term such a model the Prism Model, and describe conceptually how single domain assessments can be built up to generate the holistic description through the vehicle of a layer of Exemplar Cases. The model is theoretical but future work can use the framework and principles to generate scalable and adaptable applications that can unify and improve the precision of serial measurements that integrate environmental and physiologic influences to improve the science of child health measurement.

## Review of Existing Health Frameworks and Preliminary Recommendations for Measurement in Lifecourse Health Research Studies

The central task for the Lifecourse Health Science Working Group (LHS) was to develop a model of health measurement and a methodology to guide development of a framework for measurement in the National Children's Study (NCS), one based in science and informed by existing health frameworks. Science is a process designed to achieve understanding. All of its methods involve measurement. Since its inception, the LHS recognized the integral relationship between the meaning of health and its measurement. How we as a society conceptualize health is intimately bound to scientific measurement. As a longitudinal cohort study, the NCS, by design, was to involve diverse types of data elements assessed at multiple different developmental time points that, together, would create a comprehensive measurement strategy from birth to age 21 years. These data would involve elements such as parent and child reports on questionnaires and interviews, direct assessments of anthropometric measures, biologic samples, and observations of children's various environments including homes and school settings. In addition to allowing assessment of inter-individual differences, measurement strategies in longitudinal studies of health, such as the NCS, need to account for normative processes of growth and aging, as well as changes in people's environments as they age. Because growth and developmental changes occur at a faster pace in childhood compared to adulthood, particularly during the 0–3 and adolescent years, harmonizing assessments and their meaning as children grow and mature was a particularly important challenge for the NCS and informed our thinking.

To begin our work, the LHS working group examined the language of the Children's Health Act of 2000, which mandated the NCS. Section 1004 of Children's Health Act of 2000 states that the purpose of the NCS was “*to conduct a national longitudinal study of environmental influences (including physical, chemical, biological, and psychosocial) on children's health and development.”* Based on this language, *children's health and development* are the stated outcomes of the study and the predictor variables are physical, chemical, biological, and psychosocial influences. This language highlights the inherent challenges, as biological and psychosocial factors are included as predictor (independent) variables that determine (influence) health. However they also constitute some of the measures of health—in other words, how we come to understand and define health. Thus, like virtually all other studies of health, the NCS was based on tautology. This created some ambiguity among the NCS domain measurement working groups and the LHS as to what constitutes health outcomes and what are the factors that influence health, hereafter referred to as “drivers.” This is easily seen in terms of health problems that exist along a continuum of severity and become recognized as a “disease” only after a threshold is crossed. For example, Body Mass Index (BMI) and blood pressure are both biological factors that influence risk for obesity and hypertension but they are also the metric by which we define these outcomes. Here, as in other areas, measures of “influence” are synonymous with outcome measures.

Later in the Children's Health Act of 2000, in part b of section 1004, the language changes to indicate that the NCS is a study of “child health and human development,”. “health and developmental processes,”.and “children's well-being.” This language highlights another challenge in how child health is measured health measurement. There is no conceptual clarity as to the separation between health and development in childhood and adolescence, nor is there conceptual clarity regarding the meanings and distinctions between health and well-being. For example, the most widely recognized definition of health is that of the World Health Organization (WHO), which defines *health* as a “state of complete physical, mental and social *well-being*, not merely the absence of disease or infirmity” (1). This definition suggests that health and well-being are synonymous. Further, these terms—health, and well-being —are in common parlance and are widely used throughout the world, although the meanings differ based on the person and context in which they are used. The same issues relate to the term “development.” People believe they know the meaning of these words even though there is no true consensus. Such a quagmire is especially challenging for measurement, and particularly that which is required for a longitudinal study.

This paper cannot provide definitive answers to this conceptual complexity. Instead, we provide a brief review of conceptual approaches to health and provide several recommendations for measurement considerations related to longitudinal studies. Following these recommendations, this paper presents a new multi-dimensional model of health measurement, the PRISM model, developed to provide a conceptual basis for health measurement and outlines a systematic approach using Exemplar cases to link the domains of the PRISM Model to specific measurement plans at each assessment point for longitudinal research.

### Conceptual Approaches to Child Health and Health Development

The unique opportunity of the NCS focused us on health and the development of health (referred to as *health development* throughout this document). While this sounds like a straightforward task, the LHS recognized that the vast body of medical science is based on understanding disease, although it is often referred to as “health research.” To fulfill the goals of the NCS and assess health and its development, we reviewed selected prior definitions and models of health in order to clarify the conceptual basis for our work and explored the concept of human development from a measurement perspective.

Human development is a process that can be understood from different perspectives and at different levels. The process of child development is often viewed as the outcome of an orderly series of changes in structure and function. In recent years, with development of the field of neuroscience, biomedical and psychological developmental research has become much more integrated. However, these fields focus on individual level processes. Although individually focused, Bronfenbrenner's highly influential Bioecological model of human development broadens our perspective to include the multiple types of contexts and levels of environmental influences by situating the child in his/her social contexts, a context comprising nested environments from the most immediate micro-environment to ever more distal, but still influential environments at the meso- and macrosystem levels (2, 3).

One of the most widely known and broadly applied measures of human development is the Human Development Index (HDI) of the United Nations Development Programme (4). The HDI, which operates not at the individual level, or even the meso-system level, but at the macro level of the country in which an individual resides, is a widely used paradigm that focuses on people and their capabilities as a means to expand beyond conventional financial measures for assessing governmental policy and investment priorities. The HDI has similarities to definitions and models of health, in that it is both multilevel and multi-dimensional. Health is one of its three dimensions (standard of living, knowledge, and health).

However, the way health is operationalized in the HDI is severely limited in that health is equated with life expectancy at birth. Nonetheless, it is noteworthy that a societal-level measure of development is firmly centered on the health of the population, underscoring the pervasive connections between health and development.

The inadequacy of the HDI's single measure of life expectancy to define health is clear. As previously mentioned, the most widely recognized definition of individual-level health continues to be the World Health Organization's definition from 1946: a “state of complete physical, mental, and social well-being, not merely the absence of disease or infirmity.” Although extremely influential to this day, this definition conflates health with well-being. Furthermore, the requirement for “complete” well-being is utopian and cannot be achieved (5). Such a definition also suggests that those with congenital anomalies or disabilities could not achieve health, sets parameters on the potential variation in normal health, and creates a false dichotomy that one is either healthy or unhealthy.

A somewhat clearer understanding of health was posited by Kuh et al., who maintained that it is “The accumulation of biological resources, inherited and acquired during earlier stages of life which determine *current health* and *future health potential*, including resilience to future environmental insults (6).” This clearer statement of current health also adds a future orientation and brings in the notion of health potential. Kuh et al. specifically address the predictors of health in early life, including parenting skills and style, family functioning, and role modeling. However, the genesis of this framework was intended to explain pathways to adult health, particularly in relation to chronic disease epidemiology, not patterns of development of infant, child, and adolescent health. Another important health framework explaining adult health differences was that of fetal programming (7). This framework highlights critical and sensitive periods in development, including maternal and paternal preconception health, pregnancy, and prenatal development. However, in relation to child health, the fetal programming framework has been criticized as overly deterministic, particularly in relation to the multilevel, multi-factorial nature of today's burden of chronic diseases. A more accurate characterization of the role of fetal growth and development is that of fetal conditioning (8). With the exception of studies on birthweight and the obesity epidemic, the fetal programming/conditioning framework has yet to become a major focus in child health studies.

The life course health development model provided a conceptual advance by integrating a developmental perspective into the concept of health, and describing how health development is applicable to both individuals and populations (9). The model is built on the recognition that although early determinants, exposures, and influences may not become phenotypically evident for years or decades, such early events can lead to later consequences. These include life expectancy as well as performance of such functions and capacities as cognition, mood, physical activity, growth, and fertility that influence the development of disease, disability, and dysfunction, as well as societally valued outcomes such as readiness for learning and school and job performance. Moreover, health risks and disease conditions evolve over time, co-exist with positive health states, and can be mitigated or exacerbated by social, physical, and biologic contexts (10–15). Each person's life course health development continues as it gains complexity through the interactions of multiple components across time. For example, an exposure at one age and stage of development may lead to multiple consequences depending upon subsequent exposures, modulation, and contexts.

Looking across these human development and health frameworks, several commonalities are apparent. At the most basic level, there is a focus on person-level and system functioning. The health frameworks also focus on emotions, perceptions, cognitions, and behaviors. These processes seem to become the purview of the “health” field in contrast to the human development field only when there is dysfunction, highlighting the predominant focus on disease and negative aspects of health. It appears that despite the perspective of the WHO definition that health is a positive state (“complete sense of physical, social and emotional well-being”), positive health has been integrated with “development,” whereas health has typically been measured in terms of disease and dysfunction or their absence.

In addition to typically focusing on disease and disability, until 2004, “health” was basically synonymous with adult health. Little attention was paid to children's health (16–36). Recognition of this lack of attention laid the foundation and provided the rationale for the 2004 Institute of Medicine (IOM) report*, Children's Health, The Nation's Wealth*. This landmark report developed a definition of children's health that built on the 1986 Ottawa Charter for Health Promotion, which was instrumental in advancing the health promotion movement posited that, “Health promotion is the process of enabling people to increase control over, and to improve, their health (37).” To reach a state of complete physical, mental and social well-being, an individual or group must be able to identify and realize aspirations, to satisfy needs, and to change or cope with the environment. Health is, therefore, seen as a resource for everyday life, not the objective of living. Health is a positive concept emphasizing social and personal resources, as well as physical capacities. The Ottawa Charter did not include biology or physiology in its conceptualization of health promotion and indeed, defined health promotion, not health. The IOM report claimed that a specific definition for children's health, separate from adult health was needed in order to account for “their special characteristics, particularly rapid development during childhood. and must also consider multiple influences that interact over time in different ways as children develop and change.” The 2004 IOM report defined children's health as “the extent to which individual children or groups of children are able or enabled to a) develop and realize their potential, b) satisfy their needs, and c) develop the capacities that allow them to interact successfully with their biological, physical and social environments.” The IOM report attempted to clarify the relationship between well-being and health, stating that well-being is an individual's self-appraisal linked to quality of life, fulfillment and “ability to contribute to society and one's own family.” The Committee went on to state, “The Committee contends that behavioral, psychological and social well-being are core aspects of health and has incorporated these in the domain of “health potential”.” This definition is the closest to what the mandate of the NCS articulated, focusing on positive health, rather than the absence of disease, and considering health potential as a core construct. The IOM definition is consistent with a living systems perspective on health, such as that articulated by Forrest (38–42).

Finally, given the NCS focus on the health of populations of children, it is worth mentioning at least one -of the current definitions of population health. In 2003, Kindig and Stoddart defined the term “population health” to mean the measurement and distribution of health outcomes in a population and the patterns of determinants that influence such outcomes, as well as the policies that influence the optimal balance of determinants (43–47).

## Recommendations

Based on this review of selected, influential frameworks and definitions, the LHS Working Group addressed the following two questions related to measurement and in doing so, developed recommendations regarding health measurement for longitudinal life course studies to guide our future work.

What are major challenges impeding advances in health measurement? Many of the conceptual and analytic challenges that life course longitudinal health research is facing today are due to the recursive and multi-dimensional nature of the relationships among health, development, well-being and the environmental contexts in which an individual exists. As we age, outcomes become predictors which then become future outcomes. The recursive and reciprocal nature of these relationships is present within and across levels. For example, low socioeconomic status (SES) can increase chances of a child's exposure to lead and having low calcium levels, placing her at increased susceptibility (i.e., lower biologic adaptability) to lead toxicity and at increased chance of neurodevelopmental impacts, increased demand for caregiving, and financial burden on family. At the individual level, poor health can influence and constrain behavior, sometimes with long-term consequences, such as poorly controlled asthma limiting physical activity in a child; once the asthma is under control, low physical activity can powerfully influence that child's health later in life (e.g., obesity and cardiometabolic risk). Across levels, we know that family socioeconomic status (SES) influences children's health but children's health also influences family SES. For example, though we often associate social causation with regard to health disparities, a child with special health care needs or severe chronic illness can cause downward social and economic mobility for a family (48–52). In this case, the child's poor health influences the family SES which, in turn, can impact the health of all family members, including future generations, over time. Our current conceptualizations do not recognize or address this iterative, recursive nature, but instead inherently assume linearity.

## Recommendation 1: The LHS Group Recommends That New, Non-linear, Recursive Health Models Be Developed, As Well As the Necessary Analytic Methods Needed for Life Course Studies

Are health and development separate constructs or are they integrated in health development? The LHS Working Group debated whether health and development should be recognized as separate constructs or should be subsumed into health development. The values of both perspectives were explored. Ultimately, the importance of making a distinction between health and development for conceptualizing and developing interventions led to the group to adopt the position that health and development remain separate constructs. The need for distinguishing health from development was highlighted with this example:

An infant with motoric delay is seen in a primary care office. If the cause of the motoric delay is constant swaddling and carrying in papoose, then intervention is parental education. This is a developmental intervention. If the cause is neurologic/genetic, the child needs medical assessment and care, a medical care intervention.

As the example illustrates, developmental problems that are health-based need medical care intervention but developmental problems that are not health related, and instead are largely environmental—require a fix that is extraneous to the child and therefore may not be appropriate for the medical care system. Developmental interventions are more likely to involve social systems or changes in the physical environment. However, the importance of structural changes to improve health outcomes is a critical issue that needs to be more clearly articulated.

Importantly, the concept of “health development” is useful and distinct from its embedded terms. Health development is the patterning of the changes in health that occur over time, due in part to maturational processes and in part to the interaction of the biologic and personal health characteristics with environmental factors. Health development highlights the time dimension and the dynamic nature of our lives, which has often been lacking in health measurement. Models of health development have to be able to incorporate and demonstrate health for those with both short and long lives, as well as typically developing and non-typically developing children.

## Recommendation 2: Health and Development Should be Recognized As Separate Constructs, and ‘Health Development,’ Is, Itself, a Separate Construct. All Three of These Constructs Require Further Delineation and Further Development of Measurement Strategies and Frameworks

### Summative Recommendation

New models of health and associated health measurement that recognize the non-linear, recursive nature of the dynamic interactions of each person with his/her environments are needed to better characterize the complexity of health and to catalyze the field of life course health development.

## Addressing Recommendations by Proposing a New Health Measurement Model

Overall, our recommendations highlight the critical need for long-term, longitudinal cohorts that begin early in life and extend into adulthood in order to understand life course health development. Studies aiming to address this need should develop cohorts that are based on a systematic assessment of metrics and measures of health and development as well as their physical, social, and biologic environmental drivers. In relation to the NCS, which was the focus of the LHS' work, our preliminary work highlighted the need for measurement models and practical methods that can guide and link health to measurement in longitudinal health studies.

The LHS conceptualized and developed a new measurement model of health—the PRISM Measurement Model to advance longitudinal life course health research studies. A basic assumption of the PRISM model is that health has a dimensional structure. This assumption is based on the prior work of the HMN, specifically its draft typology, and the recognition that a dimensional structure aids the development of measurement frameworks and systems.

The LHS came to consensus on four dimensions of health, all of which occur from the level of the cell through the organ and system levels to that of the individual or whole person level. These four dimensions interact at each level of a person's system organization as well as across systems. In longitudinal studies, the status of these proposed dimensions would be derived from multiple individually assessed items that are mapped to a hierarchy of concepts and domains. The specific measures used to assess the health dimensions would need to be scaled so that they are comparable across contexts and age. The LHS envisioned that consideration of these dimensions and further work on measurement of the dimensional structure of health would foster increasingly sophisticated approaches to life course health science. Our current, working definitions of the four dimensions of health are:

**Experience:** The impact of the environment, from a molecular to a system level, on the individual.

**Performance**: What the individual is able to do; level of function.

**Adaptability**: Two definitions were considered: (1) An individual's expression of a different functional state as a result of a perceived change in the environment. Perception is not limited to conscious thought. (2) An individual's capacity to respond to internal and external changes to return to baseline or achieve a new baseline.

**Potential:** What the individual may be able to accomplish if challenged at the moment or in the future. The concept contains latent functionality.

Each definition is applicable **across** multiple levels from the molecular to the system level to the level of the whole person. Measurements may occur at higher and lower levels. On a conceptual level, for this presentation of the PRISM measurement model, they are integrated at the level of the whole child.

Based on this four-dimensional structure, the PRISM measurement model defines the following health- related terms for longitudinal research:

1. **Health plane** = The resultant state of an individual at the moment of measurement, based on the measurements that define the four dimensions of health (experience, performance, adaptability, and potential) at a single point in time.
2. **Health development** = The emergent expression of an individual's health from birth to the current point of measurement
3. **Health phenotype** = The observable expressions or manifestations of health development across the life course. Health phenotypes can be assessed at any point in time in the life course, although an individual's complete phenotype will only be apparent when described from birth through the end of life.

**Figure 1:**
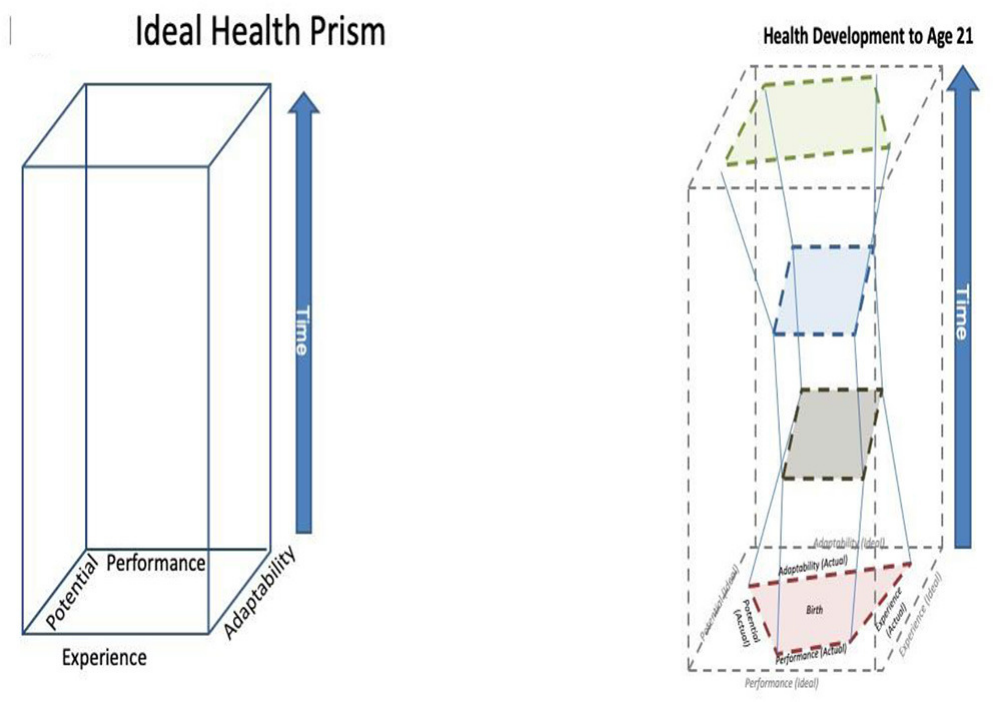
The four dimensions of health measurement form a planar shape that evolves through the dimension of time. If all four dimensions are balanced, the plane can be represented by a square and the temporal trajectory would be a quadrilateral cuboid, shown on the left. A feasible trajectory of an actual person is on the right showing changes in proportions over time.

We have termed this the PRISM measurement model because it has a geometric analogy– specifically a prism, which has volume. The conceptual foundation of this model is an *Ideal* Health Prism, which is characterized by the measured four dimensions of health, each at its “full” or ideal value, forming a square. For any individual the base of this prism is a quadrilateral whose area is determined by the measure of the four dimensions of health at birth. Time is the vertical dimension in this representation and creates the height of the prism. Over an individual's life course, the prism continuously develops, layering health planes one on top of the other over time, thereby evolving its full form, the complete health phenotype. For clarity and simplicity in this report, the LHS assumed the four health dimensions are equally weighted. With this assumption, the Ideal Health Prism would be a rectangular prism whose side measures area square based on the four dimensions and whose height is determined by the length of the life course, as seen in Figure 1. We highlight here that the assumption of equal weighting of the four dimensions needs to be thoroughly evaluated and tested.

With these assumptions, at any one point in time (or measurement), an individual's health plane will be a cross-section in the geometric prism form. As noted above, in the *ideal* health prism these health planes would always be a cuboid with a perimeter of the same size throughout the life course, as shown on the left panel in Figure 1. However, because the ideal is, by definition, unachievable, a person's health planes will always be irregular quadrilaterals.

Conceptually, this model enables us to propose new ways of characterizing an individual's health and health development. To explain the potential of the PRISM measurement model we make an analogy to the Gini coefficient, a well-known measure of inequality. The Gini coefficient, characterizes the income distribution in a country using mathematical models based on 2-dimensional geometry, specifically the area under a curve. The basic idea is that the Gini coefficient compares incomes across all members of a society. If there is no income inequality, that is, everyone earns the same, then the difference between the ideal (total equality) and the actual is zero. For the PRISM Measurement Model, this would be analogous to someone achieving the state of ideal health; there would be no difference between the actual measured dimensions of health and the ideal dimensions. In reality, some difference will always exist, so that at any point in time, the difference in surface area between the ideal health quadrilateral and a person's actual health quadrilateral is a measure of that individual's health. We have termed this measure the “Health Coefficient (HC),” which is the actual health plane surface area divided by the ideal health plane surface area. Values closer to 1.0 indicate greater health. Health development, which brings in the time component, as well as the dynamic, emergent quality of health, can be described as the difference in volume between the ideal health prism and the volume of the actual health prism created through successive health measurements. We have termed this conceptual measure of the ratio of volumes between the actual and ideal health development prisms the “Health Development Coefficient (HDC).” As with the Health Coefficient, values closer to 1.0 indicate better life course health. Thus, the “Health Coefficient” is an instantaneous value describing the differences in area between two surfaces, and the “Health Development Coefficient” is a value describing differences between two volumes.

The PRISM Measurement Model is illustrated for an individual in the right hand panel in Figure 1. The right hand panel represents examples of an individual's health development and the resultant prism derived through four health assessments at different periods in the life course up to age 21. This was the time period the NCS was to have covered. The four health assessments (study visits) are each represented by a quadrilateral plane (red = birth, brown = early childhood, blue = middle childhood, green = age 21). These quadrilaterals, which represent health at the time of measurement, are nested within the ideal health prism (gray outer boundary). Note that throughout, the actual health planes at each visit have a smaller and more irregular surface area than the ideal quadrilaterals that exist in the same plane. For this individual, between birth (red quadrilateral) and early childhood (brown quadrilateral), there were changes within the four dimensions of health, leading to slightly worse health overall, reflected in a decreased surface area of the quadrilateral. Health stabilized for the most part through middle childhood (blue quadrilateral).

Between middle childhood and adolescence/young adulthood (green quadrilateral), health improved, reflected in the increased size of the quadrilateral shape, larger even than the red quadrilateral at birth. Thus, from birth to 21, this individual's health development could be characterized as “Worse before Better.” This example is chosen to highlight the important concept that all of these dimensions are malleable and that not all are optimized at birth. For example, adaptability can increase or performance can improve, particularly during critical or sensitive periods of development. An example is learning self- regulation skills which increases adaptability for the socioemotional domain. Whether or not a child uses those newly learned skills would impact performance. Another example could be a child who develops asthma and is treated appropriately, allowing for control of the condition and the ability to improve in all dimensions. This highlights that as a general principle the larger the surface area of the health plane, the closer the person is to ideal health; the smaller the surface area of the plane, the further the person is from ideal health. All planes are parallel to the base.

**Figure 2:**
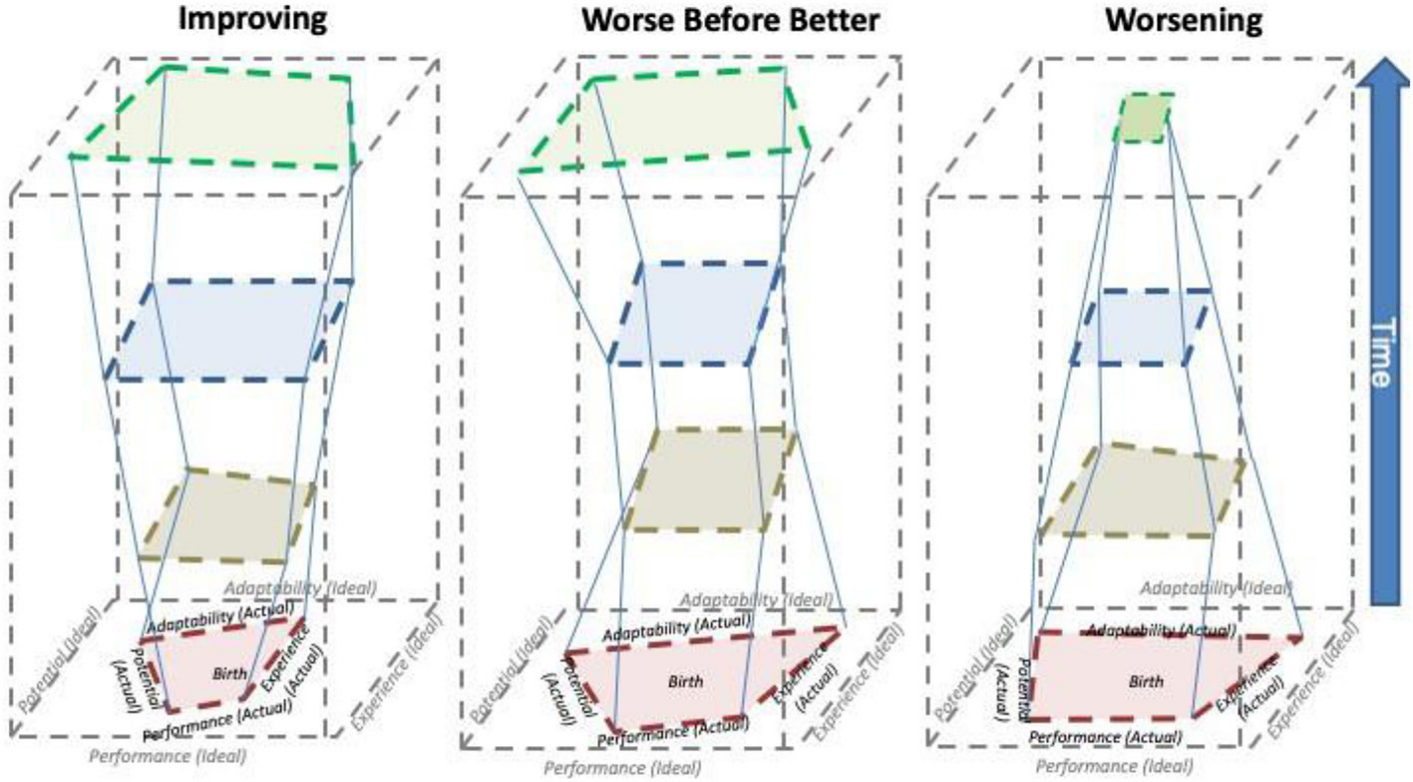
On the left, Improving phenotype is represented by the prism in the shape of an inverted trapezoid, with its resulting volume. An example would be a premature infant with lung disease that normalizes over time with effective intervention. In the middle, the Worse before Better phenotype is represented by an hourglass shape. An example may be a neurological condition that deteriorates until an effective intervention is developed that reverses the trend. On the right, a Worsening phenotype is represented by a pyramidal prism. An example of the Worsening phenotype would be a chronic, multisystem disease diagnosed in infancy such as Tay-Sachs disease that is not amenable to new therapeutic options.

The Health phenotype derives from the cumulative expression of health development over the life course from birth to death. The height of the prisms reflects the length of the life course. The shapes of the prisms created over time in a population, while unique to each individual, will follow some general patterns. Three phenotype examples are shown in the Figure 2. On the left, Improving is represented by the prism in the shape of an inverted trapezoid, with its resulting volume. An example of the Improving phenotype might be a preterm infant with mild broncopulmonary dysplasia (BPD) who gains pulmonary function throughout early childhood and adolescence. In the middle, the Worse before Better phenotype is represented by an hourglass shape. To illustrate the “Worse before Better” phenotype, take the example noted previously of a heavily swaddled but otherwise healthy infant with motor delay due to restriction of movement. With intervention, improvement in all four dimensions of health is likely and this child's overall health would improve. On the right, a Worsening phenotype is represented by a pyramidal prism. An example of the Worsening phenotype would be a chronic, multisystem disease diagnosed in infancy, such as cystic fibrosis that isn't amenable to new therapeutic options. For a healthy child whose changes in the dimensions of health are normative, a stable phenotype would be seen, similar to, but with less volume than, the ideal health prism (this prism is not shown). As noted above, health phenotypes can be assessed at any point in time in the life course, but due to the dynamic nature of health as we age, the complete phenotype will only be apparent at the end of life.

The LHS also considered how the PRISM Measurement model might be useful for exploration of health differences at the group or population level. To date, this work is purely conceptual. For example, as noted above, average life expectancy at the national level is the measure of health used by the UN's Human Development Index for cross-national health comparisons. A national average Health Coefficient (HC) or Health Development Coefficient (HDC) could be an analogous population level measure for use in health surveillance or comparison studies. For example, in healthier societies, on average, health at birth would be close to the ideal health prism, so the HC would be high (close to 1.0, a small gap between ideal and actual), reflecting good population level health. Many measures used to compare health across nations focus on birthweight and infant mortality. Differences in the HC at birth may provide a more integrated measure to compare health across nations. Furthermore, the typology of health phenotypes within a population may help elucidate mechanisms underlying health disparities both within and across countries, and cross-country differences in the HDC could provide clues to overall population health disparities.

As noted above, further work is needed to link the PRISM model to measures and derive strategies to accurately assess the four dimensions of health. Some of this conceptual linkage work began in the LHS. The early stage of this work is explained below in the Exemplar Case approach section.

Finally, it is important to highlight an important lesson from geometry with regard to conceptualization and measurement of health based on the PRISM measurement model. When any quadrilateral figure is constructed, one of the dimensions may need to be constrained. That is, once three of the four sides are determined, the last one may be constrained to exceed a certain level in order for the shape to close.

For children who are healthy at birth and typically developing, performance may likely be the constrained dimension should the need for constraint arise. For children with congenital illness or disability, potential may need to be constrained. This does not imply that such children cannot be healthy compared to typically developing children. Indeed, a strength of this model is that the four dimensions are equally weighted, such that a child with a disability who adapts and through experience and a supportive environment learns how to perform at a very high level may have better health at the level of the whole child than a child without a disability who has greater potential, but less adaptability and lower performance.

## Relationship of the Prism Model to the NCS's Domain Working Groups

As noted above, a basic assumption of the PRISM model of health is that health has a dimensional structure. This assumption was based on work of the NCS HMN. Prior to the formation of the LHS, the HMN, in partnership with several trans-NIH initiatives such as the Patient Reported Outcome Measurement Informations Systems (PROMIS®) and the NIH Toolbox® for Assessment of Neurological and Behavioral Function (NIH Toolbox), reached consensus on five basic tenets:

1. Health is a multidimensional concept
2. Each dimension can be assessed along a continuum from very low to very high levels
3. Each dimension includes multiple domains
4. Each domain can be accessed via multiple measurement modalities
5. Health develops over time and in response to a complex and dynamic set of child x environment interactions

The PRISM Model is consistent with these tenets.

**Figure 3:**
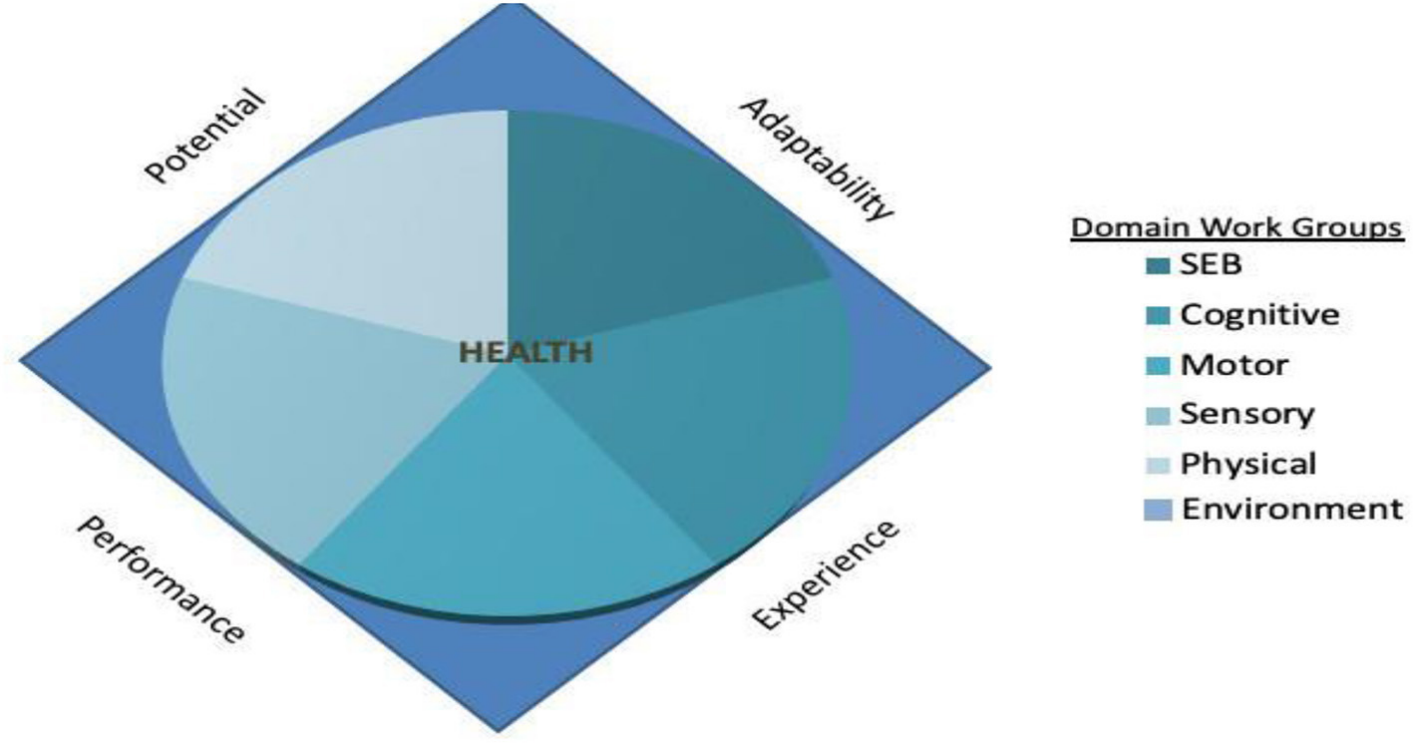
Domain Working Groups (DWG) developed specific measures in the context of the four dimensions of health measurement so that a functional continuity could be established through mapping from single measurements into higher order descriptions.

Within the NCS, the manifestations of health and strategies to measure those manifestations were the purview of the five child-related Domain Measurement Working Groups (DWGs): Social Emotional Behavioral (SEB), Cognitive, Sensory, Motor, and Physical Health and Systems (Physical). A sixth DWG focused on the environment, since the fifth basic tenet of the NCS acknowledged that health develops over time due to child-environment interactions. The relationship between a health plane from the PRISM Model's Ideal Health Prism and the other Domain Working Groups measurement plans and strategies at the corresponding point in time (study visit) remains as work in development. Because each dimension has multiple domains and measurement modalities (Tenets 3–4), the measurement plans and strategies are nested within the quadrilateral. The key interactional role of the environment is acknowledged by placing its measurement around the base of the health plane. Each Domain Working Group assessed options, made selection, and in some cases developed or proposed new measurements to capture specific age related assessments. The results of these efforts are contained in a series of monographs that are companions to this one. A representation of these relationships is shown in Figure 3.

## The Exemplar Case Approach: Linking the PRISM Model to Measurement of Drivers and Direct Measurements

This section describes the development and utility of the Exemplar Case approach, a measurement identification strategy that can be used to prioritize measures and prevent potential gaps in measurement in longitudinal, life course health development research studies such as the NCS. The PRISM model of health measurement differs from prior efforts in several ways, particularly in that, conceptually, it can be linked to discrete sets of measures. For the NCS, the LHS planned this linkage as a multi-step process in which the dimensions of health are first mapped to a limited series of Exemplar Cases.

Exemplar cases illustrate a range of person-level characteristics that are likely to be manifest in a healthy 21-years old. By design and nature a longitudinal cohort study until age 21 should be able to explain the factors and processes that lead to the development of these exemplary cases, as well as to other health states. Each Exemplar Case describes a characteristic of a 21-year-old person, the age NCS study participants were to have obtained at the conclusion of their involvement with the study. The nine Exemplar Cases are a set of person-level functional capabilities that result from the dynamic interactions between a young person's biology (genetics, anatomic structures, and physiology) and psychosocial and physical environments over the course of development. Exemplar Cases are mapped to a series of drivers selected on the basis of theory, expert opinion or empirical evidence because of an expected relationship between each driver to a specific Exemplar Case. Different drivers are identified for different age/developmental periods (assessments). The relevant drivers at each age (assessment point) can then inform the specific assessments needed for longitudinal studies, which can be obtained via questionnaires, quantitative measurements of physiologic function, structured observations, or other modalities. Once the battery of assessments is relatively complete for a given assessment visit/contact, the constructs being assessed can be cross-walked with the drivers to ensure that there are no gaps in the measurement of relevant drivers.

**Figure 4:**
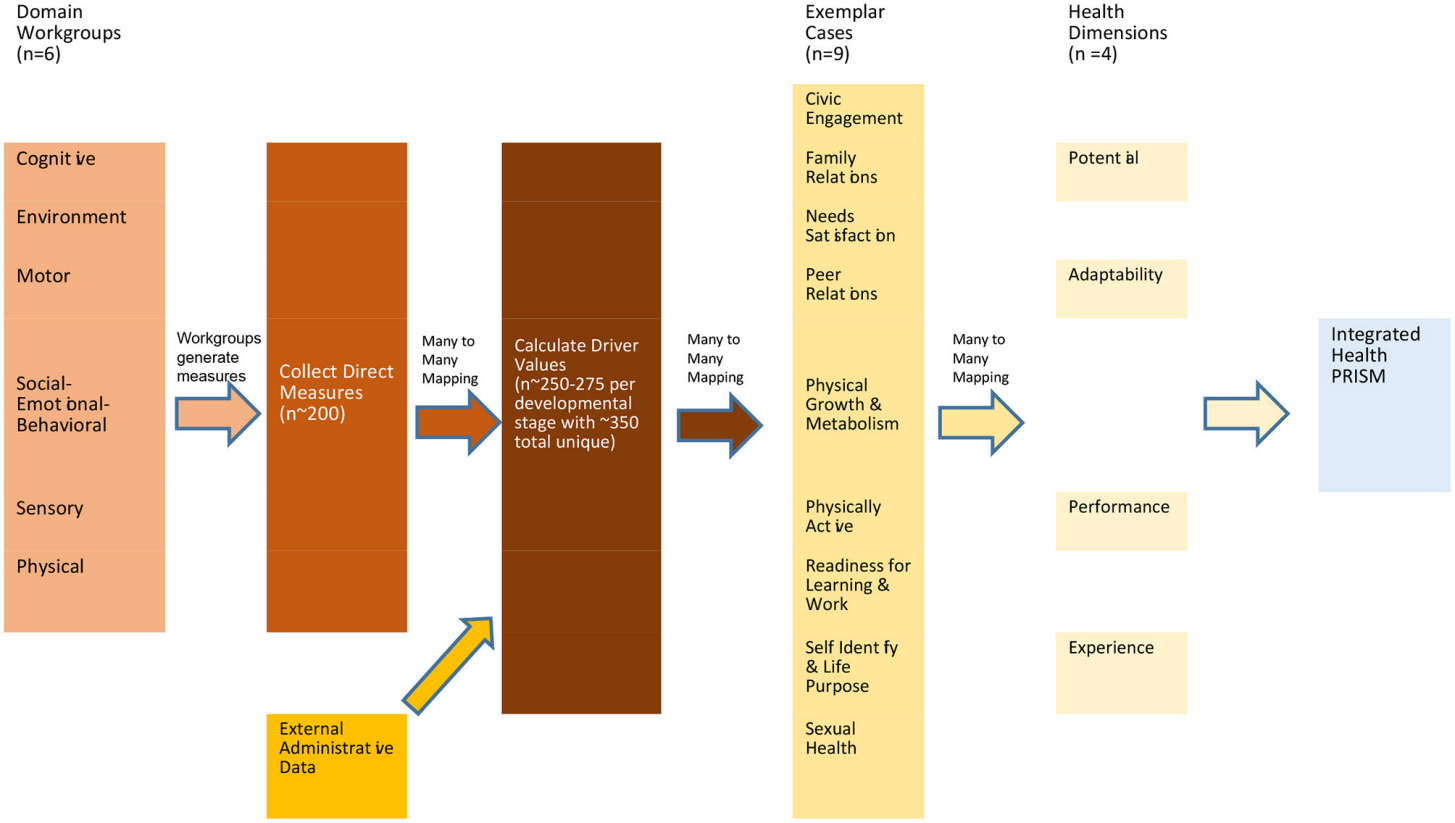
Many to many mapping among system components. The Domain Workgroups propose specific assessments that collectively form a library of direct measures that are age and developmentally appropriate across all six of the assessment domains. From the library of unique assessments, a visit schedule is generated so that each visit is as complete as feasible but not burdensome. The output from the direct assessments is combined with external administrative data and mapped in a series of many to many relationships to the catalog of drivers. Once values are determined for the relevant drivers, those values are subsequently mapped via a many to many process to the Exemplar Cases, which are in turn mapped to four health measurement dimensions.

The overall schema is illustrated in the schematic that is Figure 4. The Domain Workgroups propose specific assessments that collectively form a library of direct measures that are age and developmentally appropriate across all 6 of the assessment domains. From the library of ~200 unique assessments, a visit schedule is generated so that each visit is as complete as feasible but not burdensome.

The output from the direct assessments is combined with external administrative data and mapped in a series of many to many relationships to the catalog of drivers. Once values are determined for the relevant drivers, those values are subsequently mapped via a many to many process to the Exemplar Cases.

The Exemplar Cases, once populated with values representing a temporal snapshot of an individual, can then be in turn mapped using a many to many process to the four health dimensions. The values of each health dimension for an individual can then be compared to an ideal health dimension to generate a ratio that can be used as a benchmark for comparison with past and future values to evaluate trends.

Note that in the many to many mapping process, an output can be relevant and mapped to become an input for several components of the next layer. Thus a single output from a direct measurement can be relevant to and provide input for several of the drivers. The value of a single driver can be informative to and map into several of the 9 Exemplar Cases, and the Exemplar Case outputs, in turn, can inform any or all of the 4 health dimensions.

## Further Description of the Exemplar Cases

The criteria for identifying an Exemplar Case are that it describes.

- an example of expectations for a healthy, well-functioning 21-year-old
- the manifestation of a set of integrated dimensions of health and development
- a set of characteristics that evolve during childhood and adolescence

The currently identified nine Exemplar Cases are:

1. Civic engagement
2. Family relationships and caregiving
3. Needs satisfaction
4. Peer relationships
5. Physical growth and metabolism
6. Physically active
7. Readiness for school/Learning/Work
8. Self-identity/Life purpose
9. Sexual health

These nine cases are not intended to be exhaustive but instead are illustrative of how a range of important person-level functional capabilities that society expects of healthy young persons can be utilized to guide measurement selection. It is noted that both early life biologic endowment and sufficient environmental opportunities are necessary for these capabilities to develop. Both of these areas were critical aspects of study in the NCS. The Exemplar Cases developed by the LHS reflect capabilities identified in prior models of health by the LHS's review, including the WHO's definition of health (peer relationships), Kuh's notion of health capital (family relationships), the Ottawa Charter (needs satisfaction), the 2004 IOM report (civic engagement, self-identity/life purpose), and the Life Course Health Development Framework (Readiness for School/Learning/Work, Physically Active, Sexual Health, and Physical Growth and Metabolism).

Young adults will differ in the extent to which they have developed each of these positive characteristics. Additional Exemplar Cases could be developed in the future to capture additional characteristics. While we present only one case that is overtly physiologic, Physical Growth and Metabolism, and one case that is overtly related to behavior, Physically Active, physiologic functioning and behaviors are integral to all cases. Of note, while the Exemplar Cases reflect aspects of a healthy, well-functioning 21-year-old, they are presented in a neutral manner, without a value label of positive or negative, so as to support measurement across the entire spectrum of health. Moreover, all these Cases are manifest earlier in life than age 21, as they develop over the period of childhood and adolescence.

Underlying the Exemplar Case approach are the assumptions articulated earlier in this paper, that health risks and disease conditions evolve over time, co-exist with positive health states, and can be mitigated or exacerbated by social, physical, and biologic contexts. Thus, the case approach is applicable to all youth, including those with manifest disease or disabling conditions as many young people with chronic illness or disability can demonstrate characteristics of healthy functioning.

As described below, the Exemplar Case approach can help describe the factors that contribute to achievement of these integrated states in a 21-year-old through the identification of likely early life predictors of these outcomes. These predictors can be referred to as “drivers,” factors that influence the phenotypic expression of an Exemplar Case.

**Figure 5:**
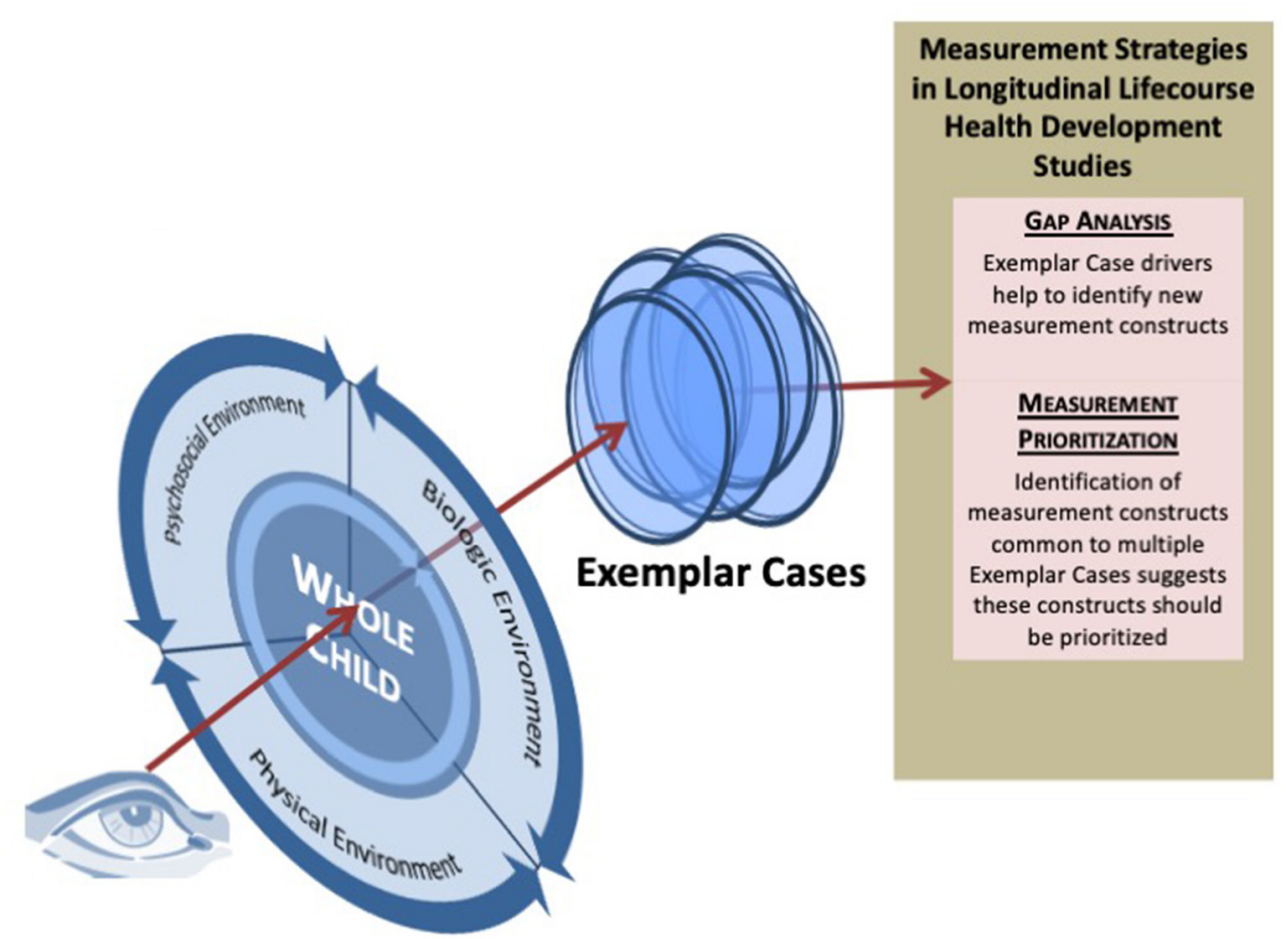
Depicts a perspective on the Exemplar Case approach. The focal lens represents the influence of the interacting biological, psychosocial, and physical environments. Over time, their dynamic interaction results in the overlapping lenses of nine Exemplar Cases. These lenses support an approach to measurement that requires determining the biological, psychosocial and physical drivers that interact to predict each of the nine Case outcomes at age 21. This measurement strategy provides the ability to prioritize measurements and identify potential gaps in measurement.

Figure 5 depicts a perspective on the Exemplar Case approach. The focal lens represents the influence of the interacting biological, psychosocial and physical environments. Over time, their dynamic interaction results in the overlapping lenses of nine Exemplar Cases. These lenses support an approach to measurement that requires determining the biological, psychosocial and physical drivers that interact to predict each of the nine Case outcomes at age 21. This measurement strategy provides the ability to prioritize measurements and identify potential gaps in measurement.

The definitions of each Exemplar Case are presented in Table 1 at the end of the paper. Table 1 also provides a description of each case in terms of the PRISM model's dimensions of health (experience, performance, adaptability, and potential) that are described earlier in this Chapter. The LHS identified drivers for each Exemplar Case at each of five distinct developmental periods: maternal/prenatal, infancy, early childhood, middle childhood, and adolescence. The driver lists developed by the LHS are illustrative, not comprehensive, and are used to explain the Exemplar Case approach to measurement identification. Additional drivers would be identified and added to those identified thus far through in- depth literature reviews to make these lists comprehensive and complete. Examples of these Exemplar Case Driver lists for the developmental period of Early Childhood are found in Table 2. An Appendix provides a glossary with definitions of terms.

**Table 1:** Exemplar case definitions and description by health dimensions.

		**Health dimensions**			
**Exemplar case**	**Definition**	**Experience**	**Performance**	**Adaptability**	**Potential**
Civic engagement	A person's interest, involvement and commitment to individual and collective actions designed to identify and address issues of public concern and importance.	Life activities that shape the development of an awareness of the influence of contexts on people's lives; awareness of the role of the individual in making the community better; awareness that one can make a difference outside one's home/family and awareness of the needs for improvements in one's larger environment.	Level of participation in activities to improve the well-being of others outside one's immediate home and family.	Has tenacity to persist in involvement in activities despite setbacks, changing contexts and consequences, in pursuit of actions to address needs.	Sense of commitment; understanding the value of individual and collective voices and actions as well as the impact of activities to improve the larger society.
Family relations and caring for others	Connections with parents, siblings and extended family members related to living and being together and being a part of the family. These include beliefs and understanding about love and trust among family members, the predictability of socioemotional support and help that can be received to solve problems. Also includes one's capacity, skills and interest in caring for others with dependency needs and for establishing and developing strong supportive emotional bonds.	Social, emotional and physiologic awareness of the quality of interactions with caregivers and other family members. Awareness of belonging to a family and identifying with one's family.	Child's awareness of family members' roles, and expectations of these roles relative to the child. Child's responses to and engagement with family members.	Adjusts to changing events within the family, and to changes in the roles of family members.	Extent to which child can adapt to new roles and changing family composition and to which child takes on increasing responsibility within the family.
Needs satisfaction	An integrated health capacity that allows a person to address core human needs, including Subsistence, Protection, Affection, Understanding, Participation, Leisure, Creation, and Identity. Develops over the life course and no one can satisfy all his or her needs without the assistance of others.	Awareness of physical, emotional, and social needs, of one's relative success in meeting needs and reasons for failure; awareness of barriers to achievement.	Consistent with developmental status, extent to which person identifies own needs and is able to articulate them to others, as appropriate. Extent to which person takes action to meet needs in ways that allows the maintenance of positive relationships with others. Timing and appropriateness of communication of need for assistance.	Is aware of context and environmental barriers to need fulfillment. Has the ability to adjust and delay gratification of needs; has ability to modify approaches to need fulfillment, adjusts to environmental limitations or barriers.	Capacity for awareness, for managing drives and desires, and ability to plan to meet foreseen needs and adapt to needs arising from environmental or developmental changes.
Peer relations	Social connections, including intimate partners, with whom a person spends time, both in person and virtually, and with whom he or she engages in leisure, learning or work activities.	The awareness of and participation in repeated interactions with people outside the immediate family. One's early social and emotional interactions with caregivers shape emotional and physiologic responses that provide the foundation for all relationships, including those with peers.	Level of engagement in activities with others of similar age or interests, who are considered friends and associates in various environments such as the neighborhood, school, sports, and work. This includes connections through emerging technologies.	Demonstrates persistence in maintaining relationships with significant others who are friends or meaningful associates despite challenges to the relationships.	Capacities for social interactions; age-appropriate skills to effectively engage others and maintain relationships over time; extent to which one values social connections and sense of belonging to a group.
Physical growth and metabolism	Changes in size, body habitus, and metabolic functioning over time. Physical growth and metabolic functioning are direct indicators of health, involving utilization of energy and biomaterials for structural growth, renewal, remodeling and adaptation. Life experiences and environments influence growth trajectories, metabolic functioning, and the tempo of growth, affecting both present and future health.	Exposure to environmental demands in the context of nutritional and psychosocial environments and genetic predisposition that result in changes in body size, mass and metabolic functioning.	Anthropometric and functional increases that are aligned with age calibrated norms and trajectories.	Maintenance of anthropometric trajectory with changes in the environment. Adjustment of energy stores based on activity, nutritional intake and composition, and environmental context.	Anthropometric and functional growth and metabolic adjustments to survive and reach human adult size and function.
Physically active	The integrated state of being physically active refers to using one's body to move in a coordinated way that supports daily functioning, exercise, play, art, or competition. It depends on the capacity to process and utilize energy along with biophysical development, sensory input, psychosocial contributors, and physical environment.	Exposure to environmental demands for bodily activity that, together with nutrition and metabolic function, serve to develop the body and support purposeful movement. Participation in activities that shape the form and function of the body through internal and external feedback, whatever the type of physical activity one engages in.	Ability to initiate and maintain purposeful, coordinated movement necessary for daily living and to achieve the child's goals related to strength, coordination, movement and endurance. Performance occurs at every level from cellular, to organ, system and the whole person.	Adjustment of energy stores based on activity, nutritional intake and composition, and the environmental context. Able to learn or train to adjust to new contexts, including illness or impairment, or setting new achievement goals.	The individual's metabolic, neural, cellular and organ system capacity to be physically active, within the context of one's physiologic maturational state and psychosocial environment.
Readiness for school, learning, and work	The physical, emotional, cognitive, language and social assets required to be ready for formal schooling, whether given at home or through public or private schools, and for the capacity for lifelong learning readiness. Capacity, skills and interest to contribute economic and personal value to an enterprise and to society as a whole.	Exposure to the foundations of health that influence the physical, emotional, cognitive, language, and social assets that support the capacities for learning. Awareness through activities and communications of the cultural value of thinking critically, being creative, solving problems, and engaging in tasks involving thinking and the production of outputs, motivating engagement in learning activities.	Consistent with developmental status, children and youth are able to engage in activities that require them to pay attention to instruction and attend to challenging tasks that involve skills such as understanding and recall, critical thinking, creativity and cooperation with others to work with new material and effectively gain and demonstrate knowledge and skills.	Demonstration of attention, perseverance, effective acquisition and production of knowledge and skills across a range of settings and situations.	Capacity to self-regulate and organize relevant cognitive schema; has sufficient intrinsic motivation to apprehend novel experiences, engage with structured teaching and foster self-directed learning leading to new knowledge and skills.
Self-identity/Life purpose	A strong and clear sense of “Who I am” and that life is purposeful and meaningful.	Social learning through observation and interaction with others as well as exploration of and engagement in relational, social, cultural and spiritual roles. Includes imaginary exploration and play involving novel behaviors and role attributes.	Displays clarity and confidence regarding one's values, goals and place in the family and community. Involves personal probing and exploring various roles through interactions with others. Capacities for evaluating the risk-benefit balance in the context of life goals.	Ability to realistically modify one's goals and behaviors based on opportunities and realities.	Capacity to develop a sense of self and life goals that are realistic, recognize others and affirms one's own identity.
Sexual health	Developmentally appropriate reproductive, motor, and sensory system functioning and cognitive/emotional capabilities that enable individuals to freely choose to engage in and enjoy sexual behaviors. Sexual health and development begins at birth, occurs throughout childhood, and continues throughout the life course.	Awareness of gender and sexual identity in the context of family, peers, and society. Level of understanding about familial, cultural and spiritual expectations regarding sexual identity and sexual behavior. Extent to which one has positive feelings and satisfaction with behavior in addressing sexual desires, without frustration or shame.	Capacity for appropriate neurohormonal and emotional response to sexual thoughts, impulses, and behaviors. Ability to modulate and regulate sexual impulses, expression of sexual needs, desires, and behaviors.	Able to control and display cultural and personal sensitivity in sexual behaviors/actions based on context. Able to cope with interpersonal, familial and societal responses to expressions of sexuality, sexual identity, and sexual behaviors.	Capacity for behavioral control of sexual impulses and control of emotional responses to such impulses.

*Time, even when not explicitly stated, is an integral aspect of all the health dimensions. The past influences current and future aspects of experience, performance, adaptability, and potential*.

**Table 2:** Early childhood exemplar case driver list.

**Developmental stage: early childhood**									
**Family relationships**	**Peer relationships**	**Growth**	**School/Work readiness**	**Needs satisfaction**	**Sexual health**	**Self-identity**	**Life purpose**	**Civic engagement**	**Physically active**
Genotype (Vaospressin, Oxytocin, Serotonin transport genes); Epigenetics	Child Stress	Epigenetics	Child-level temperament (introversion): Shyness, nervousness, fear	Physical functioning	Gender identity	Attachment	Self-regulation	Academic performance	Age
Child stress	Temperament	Race/Ethnicity	Child-level temperament (extroversion): Aggression, inattention	Balance	Gender (sex) roles	Neurocognitive development	Parent-Child Relationship	Access to community centers	Acculturation
Temperament	Security of attachment	Nutrition	Reports of psychosomatic symptoms (headaches, stomach aches)	Auditory	Relationships to adults (non-parental)	Social connections	Home environment resources	Access to green spaces	Descendency
Emotion regulation (Effortful & Reactive control)	Ego Resilience/Grit (belief in oneself, persistence in pursuit of goals)	Eating behaviors	Self-regulation	Visual functioning	Temperment (personality factors)	Peer relationships (social networks)	Cognitive flexibility	Adaptability	Race/Ethnicity
Behavior problems	Empathy	Physical function	Inclination to use skills and knowledge (enthusiasm, curiosity, and persistence on tasks)	Coping	Media use and exposure at home	Social media use	Perserverance	Blood Pb / heavy metals	Gender
Medical problems	Emotion Regulation (Effortful & Reactive control)	Weight bearing physical activity	Conduct (lying, cheating, stealing, obedience)	Executive Function	Parent sexuality	Parent aspirations/expectations	Confidence/Self Efficacy	Cultural identity	SES categories
Impairments in physical function	Behavior Problems	Participation in team sports/organized PA	Hyperactivity/Concentration	Memory	Stress	Parent occupation	Violence	Neurodevelopmental Toxicant Exposure	Parental, sibling, friend physical activity levels
Impairment in cognitive function	Self-concept	Experiences with organized PA	Recognition of letters and sounds	Spoken language	Prior sexual and/or physical abuse	Parent mental health	Physical activity	Exposure to news media	Diet/nutrition
Parents in home	Anxiety	Metabolic disease	Vocabulary development	Health literacy	Presence disability	Presence of disability	Executive Functioning	Family structure, number of siblings	Out of the country medications purchased
Family composition and structure	Depression	Chronic disease—kidney, endocrine, thyroid, sickle cell, chronic pain	Age-appropriate expressive language	Socio-economic status	Brain development (i.e., executive function)	Presence of chronic medical condition		Food security	ED visits related to sustained ability to move (bone, muscle, asthma)
Home environment (safety, space/crowding, noise, organization/chaos)	Social Competence	Immune function	Ability to recognize basic shapes	Food security	Relationship with parents	Optimism		gender identity	Health care access
Household stability	Social Problem Solving	Executive Functioning	Ability to identify colors	Housing security	Presence of chronic medical condition	Temperment		Geographic location	Hospitalizations
Maternal cognitive & emotional control capacities	Nutrition	GI function—need proper absorption of nutrients	Ability to count	Financial strain	Chronological age	Media exposure		Household income	Primary care visits; routine preventive visits; immunizations up to date.
Family beliefs/culture re: parenting	Medical conditions; frequency and observability of symptoms	Glucose homeostatis	Academic efficacy	Neighborhood resources	Sensory functioning	Psychological development as in internalizing and externalizing disorder		Introvert/Extravert	Number of specialists; number of visits
Child neglect	Disabilities	Fat metabolism	Aspiration/Novelty Seeking/Initiative		Adverse events	Employment history		Local political climate	Types of surgeries and age of surgeries
Child abuse	Family Structure & Composition	Ca/PO4 regulation	Empathy		Coping	Positive adult relationships		Neighborhood walkability	Sleep patterns and amount
Parent availability/work schedules	Disruptive life events (divorce, moves)	Stress	Social Efficacy/Inefficacy		Loneliness	Chronological age		Parental health	Exposure to second hand smoke
Parental relationship quality/stability	Home environment (space for friends, organization/chaos)	Sleep	Relationships and social interactions with adults		Parental experience of physical and sexual abuse, domestic violence	Family structure		Parental involvement	Sun Exposure
Maternal abuse/Family violence	Child Neglect	Timing of Puberty	Attachment with Caregiver		Endocrine disruptor exposure in home and community	Food insecurity		perceived number of friends	Executive function
Family adversity	Child Abuse	Physical Environment—toxins (air, soil, water, food radiation)	Relationships and social interactions with siblings/peers		Residence	Objective SES		Pesticide exposure	Visual/spatial processing
Maternal Depression & other psychiatric disorders	Parental monitoring and supervision	Food security	Behaviors: Praise		Community religious norms	Presence of disability		Public/private school	Coordination
Paternal Depression & other psychiatric disorders	School Climate	Sun exposure	Behaviors: Promotion of child development		Community EtOH and susbstance use	Presence of chronic medical condition		Religiosity / spirituality	Endurance
Serious medical problems in parents or siblings	Maternal Abuse/Family Violence	Neighborhood resources	Expectations for child (e.g., child to earn college degree, ECLS-B)		State of residence (political affiliation)	History of abuse/neglect		School attendance	Fine motor
Negative parenting (Intrusive, harsh, inconsistent)	Family Adversity	SES	Parent supervision		Media exposure	Coordination		Sibling relationships	Gross motor
Positive parenting (Responsiveness, involvement, consistency)	Maternal Health & Problems (Disabilities, Depression & other impairing disorders)	Family member BMIs	Behavioral concerns about child		Quality of the school system	Sports team participation		Social network measurements	Locomotion
Child care setting quality	Paternal Health & Problems (Disabilities, Depression & other impairing disorders)	Social Networks	Mother's education level		Tax base	Hobbies			Strength
Community safety, involvement	Paternal Trouble w/ Law		Child abuse & neglect		Community SES	Community religious norms			Pain, pain tolerance
	Negative parenting		Divorce or death of parent		Presence of after school and youth engagement programs	Residence			Proprioception
	Positive parenting (+)		Sibling or Parent Substance Use			Presence of after school and youth engagement programs			Sensory processing integration
	High Parental Involvement (+)		Parental stress/mental health			Community EtOH and substance use			Vestibular
	Family Connections and Social Support		Religiosity (parent/caregiver)			State of residence (political affiliation)			Vision
	Family Community Engagement		Foster care			Media exposure			Chemicals affecting bones, muscles, nerves
	After school program participation		Poverty/socioeconomic status			Quality of the school system			Chemicals affecting bones, muscles, nerves
	Participation in social/religious organizations		Supports for families			Tax base			Chemicals affecting bones, muscles, nerves
	Peers' behaviors		Lead poisoning			Community SES			Access to green space
	Community recreational resources		Health insurance			Neighborhood safety			Proximity/access to community centers
	Community after school programs		Access to/availability of mental and behavioral services			Endocrine disruptor exposure in home and community			Traffic, speed
	Neighborhood safety		Access to/availability of primary health care			Built environment			Crime rates, lighting, bus routes/mass transit; sidewalks
			Immunizations						Radiation
			Preschool & child care						School physical activity norms; recess; organized sports; after school activities
			Head Start						Air quality

*The table below provides examples of drivers for the early childhood stage. The lists are not comprehensive*.

## Using the Exemplar Case Approach to Guide Measurement Strategies in Longitudinal Health Research

Because the Exemplar Cases describe integrated, whole person characteristics that overlap with health states created by person x environment interactions over time, the Exemplar Case approach assists in both the development and implementation of measurement strategies for longitudinal health research studies. Key to the approach are the driver lists, which create a bidirectional link, or bridge, as shown in Figure 4, between the integrated functional states described by the Exemplar Cases and a study's measurement plans through the processes of measurement prioritization and gap analysis.

With measurement prioritization, driver lists from related Exemplar Cases help studies' develop assessment plans. Measures are prioritized if they are influential across multiple related Exemplar Cases. Because study visits are timed to capture information regarding major developmental periods, as are the Exemplar Case Driver lists, drivers can be operationalized with one or more measures taken at each study visit. For a study of overall health and development, such as the NCS, multiple and even arguably all the Exemplar Cases are relevant. More targeted studies may have fewer or even a single related Exemplar Case. After development of initial measurement plans, studies can undergo a gap analysis by comparing their assessment plans to Exemplar Case driver lists at each developmental stage. Thus, for measurement prioritization, the direction is from Exemplar Case to Study Measurement Plan and for gap analysis, the direction is from measurement plan to Exemplar Case. A useful feature of the Exemplar Case approach is that both measurement prioritization and gap analysis can occur at any point in the study's design and can be repeated. Because the processes of measurement prioritization and gap analysis are iterative, the bridges strengthen with repeated use.

As noted above, an investigator can begin to apply the Exemplar Case approach at any phase in a study's development, reflecting the fact that measurement planning is an iterative process. New Exemplar Cases can also be developed for a study and its drivers identified using a rigorous scientific literature review augmented by expert input and empirical evidence.

Once a working set of Exemplar Cases for a particular study is agreed on, measurement prioritization enables development of early assessment protocols for each study visit. One example of a prioritized measure in the NCS is maternal educational attainment, which is not only a powerful driver of young adult learning, school and work performance but also predicts positive young adult outcomes for other Cases, including Physically Active, Physical Growth and Metabolism, Need Satisfaction and Civic Engagement. Once the priority drivers for a specific assessment visit are identified, the priority drivers can be used to further inform the measures ultimately planned for data collection. This methodology provides a cross walk between age, scheduled assessment, drivers and planned measurements. Once a tentative list of the most important constructs has been identified for each assessment visit, the drivers can be cross-referenced with the measurements planned for that visit, for a gap analysis. This enables study planners to determine whether each of these prioritized drivers is being operationalized with an appropriate measurement at a specific assessment visit. Finally, this same analysis can be undertaken for each of the other assessment points. This helps ensure comprehensive and efficient assessment of positive and negative health and environmental influences using the Exemplar Case Approach.

A specific example may support the implementation of these principles. The Exemplar Case School & Work Readiness as applied to early childhood has multiple drivers including ability to concentrate, recognition of letters and sounds, ability to count, empathy, lead exposure, and experience of being read to. These drivers can be in some cases directly assessed by the study measurements in a 3–4 year old child such as the NIH Toolbox Empathy scale and the Infant -Toddler Social Emotional Assessment, a Theory of Mind Scale, Pre-School Activities Inventory, parental inquiry on home reading, and water testing. These results are entered along with the other drivers that map to the specific assessments to provide both values for the drivers and subsequently values for the Exemplar Case using the drivers.

A second example will use the Exemplar Case of Civic Engagement for an adolescent where among the drivers are academic performance, resilience, exposure to news media, school attendance, and spirituality. These can be measured by the DUKE University Religion Index, school records, Resilience Scale for Adolescents (READ), and direct inquiry of the child about media exposure. As above, these collected values are entered along with the other drives into the listings for Civic Engagement with a resultant composite assessment.

Both examples require access to the mapping schema from assessment to driver and from driver to Exemplar Case. Further work is needed to develop the mapping schema necessary to integrate the assessments.

Thus, an exemplar case is a composite characteristic of the whole person that serves as an outcome and links to the multidimensional model of health, serving as a bridge to higher order concepts. The drivers that influence its outcome can also be used to describe an Exemplar Case. The set of likely drivers are the link to a more granular level of the specific measures needed to understand the development of the Case outcome. The Exemplar Case is thus the layer that bridges between higher order concepts and the technical specifications that define specific measures.

In summary, the Exemplar Case approach provides a methodology for designing the measurements for longitudinal trials by describing and utilizing the drivers of positive and valued health outcomes in young people. The Exemplar Case approach provides a methodology for identifying predictors of health outcomes that should be prioritized for measurement, and provides the basis for conducting a gap analysis to identify drivers that are not currently planned for measurement. We recognize that some domains of drivers need to be better identified and even that additional cases may prove valuable.

Although the LHS was not able to complete the validation of this methodology during the active period of the NCS, the approach had proven useful and appears to have significant potential for utilization by future longitudinal studies. The positive orientation of the cases helps ensure that the drivers that support effective health development are identified and assessed. This methodology can help ensure comprehensive and efficient planning of measurement for longitudinal studies.

## Summary and Future Directions

The Life Course Health Science Working Group addressed two complex issues central to the planning and development of longitudinal studies of child health—how to apply what is known about the development of health to measurement, and the need for health related outcomes to guide the selection of measures. Prior approaches have relied on predicting disease or its absence and had limited functionality to describe the environmental influences on health and development, which was the goal of the National Children's Study.

The LHS review of earlier and current conceptualizations of health highlighted the value of the concept of health development, the patterning of changes in health that occur over time, due in part to maturational processes and in part to the interaction of biologic and personal health characteristics dynamically interacting with environmental factors. Although it was beyond the scope of the LHS to develop a novel definition of health, dimensions of health were described. The way that the dimensions relate to one another was illustrated in the PRISM model of health measurement. This multidimensional model illustrates how the health dimensions (experience, performance, adaptability and potential) are inter-related at any point in person's life, reflecting his/her health state, and how they can serve as predictors of future health. The actual “content” of health, such as each aspect of the sensory system, can be measured across each of these four dimensions, and across time in a child's life. The dimensions of health can be measured in multiple ways, including biomarkers and clinical assessments, genetic data, questionnaire responses, observations, and monitoring over time. Measurement of health also requires measurement of the environmental influences that are constantly dynamically interacting with every child's biologic, social, and psychological functioning.

The LHS recognized that a major challenge in studies of health was the lack of primary positive health outcomes, with the result that typically the best outcomes were simply the absence of disease and dysfunction. To address the need for a set of integrated characteristics to describe what a healthy 21-year-old might look like, the LHS developed the Exemplar Cases. The nine Cases developed are not intended to be exhaustive but instead illustrate a range of important person-level functional capabilities that society expects of healthy young persons. Each Case is defined in terms of the four health dimensions and reflects a set of integrated characteristics, for example those associated with being “ready for school,” having “family relationships” or being “physically active.” Young people will differ in the extent to which they manifest these characteristics, but importantly, people with chronic and disabling conditions are able to manifest these characteristics, reflecting that health can co-exist with disease.

As the field moves forward, the PRISM measurement model and Exemplar Case approach can also be used to frame measurement planning in future life course research studies, even to characterize the measurements available in existing virtual cohorts. The drivers x measures matrix approach could be used to identify commonalities as well as distinct differences and even disconnects between cohorts synthesi from numerous studies. In fact, the non-linear, recursive health models needed for life course analysis can provide the conceptual basis for integrating cohorts at different life stages and for analyses and interpretation of outcomes. Further work on life course models and frameworks for measuring the separate constructs of health, development and health development will be necessary to provide a complete array of tools for the design of fully integrated studies of child, adolescent and young adult health and for the effective synthesis of virtual cohorts.

To inform scientific colleagues and foster the forward direction of health measurement, the LHS recognizes the importance of publishing the PRISM model and the Exemplar Case approach. We look forward to potential opportunities for stimulating the dialogue about health measurement across diverse groups of researchers, clinicians, and other stakeholders, including parents. We are hopeful that there will be opportunities for testing the applicability of the Exemplar Cases and refining the methodology in order to create a new model for characterizing positive health outcomes in longitudinal studies of the environmental influences on children's health development. We believe this approach could be directly applied to the exploding area of microsimulations and virtual experiments.

## Conclusions

Building on the conceptual work of the past 60 years, the NCS LHS team focused on the multidimensional, dynamic and developmental nature of health, particularly child health, to provide an integrated conceptual model to advance measurement of health over the life course. The LHS recognizes that much work is needed to further specify the model and to iteratively test the Health Coefficient and the Health Development Coefficient with the application of extant data. Moreover, as science and measurement technologies advance, new options and applications of the model will emerge. We believe the proposed framework provides an integrated and interpretable heuristic approach to help the developers of longitudinal life course studies determine data collection and analysis needs in order to describe and understand an individual's or a population's health journey through time.

## Author's Note

In this paper, we provide a brief review of conceptual approaches to health used by the Lifecourse Health Sciences Working Group of the National Children's Study and provide several recommendations for measurement considerations related to longitudinal studies. Following these recommendations, we present a new dimensional model of health measurement, the PRISM model, developed to provide a conceptual basis for health measurement in the National Children's Study. Finally, we demonstrate using Exemplar cases how to link the domains of the PRISM Model to specific measurement plans at different assessment time point for longitudinal researchers.

## Author Contributions

All authors listed have made a substantial, direct and intellectual contribution to the work, and approved it for publication.

## Conflict of Interest

The authors declare that the research was conducted in the absence of any commercial or financial relationships that could be construed as a potential conflict of interest.

## Appendix: Glossary of Terms Used

Adaptation—a dimension of health—The capacity of a person or a biologic or psychologic system to respond to internal and external challenges in order to restore an equilibrium state, although the subsequent equilibrium achieved could potentially be different from the baseline.

Dimensions of Health—Four aspects of health were identified as important for measurement: experience, performance, adaptation, and potential. These measurement dimensions were also defined for each of the Exemplar Cases.

Domains of Health—The domains of health were not defined by the HMN or the LHS. However, for the purposes of measurement selection and development, five health domain measurement working groups were developed to assess the full range of child health, with a sixth measurement working group focused on the environment. The five health measurement domain working groups were organized as: Social-Emotional-Behavioral, Cognitive, Sensory, Motor, and Physical Health and Systems.

Driver—A factor that influences outcomes, here used in terms of the development of health and the Exemplar Cases (see Chapter 4).

Exemplar Cases—Examples of integrated characteristics that reflect expectations for a healthy, well- functioning 21-year-old person. Nine cases have been described, although more can be added. These characteristics evolve over childhood and adolescence, resulting from the dynamic interactions between a young person's biology and the physical and psychosocial environment. These cases provide a counter- balance to the disease outcome approach for longitudinal studies of health development. They underscore the importance of measuring the known and hypothesized ‘drivers’ or factors that influence the development of each case at each measurement period in order to ensure that the factors important to health and functioning in young adulthood are studied as well as those that lead to disease.

Experience—a dimension of health—The impact of the environment on the person, both immediate and cumulative, that occurs from a molecular to a system level, as well as at the level of the person.

Health—An integrated set of capacities (of all living things) that support monitoring and interpretation of the environment, management of energy, adaptation to internal and external environments, reproduction, and the development of capabilities that enable locomotion, communication, interpersonal relationships and thriving.

Performance—a dimension of health—The capacity and functioning of one or more biological or psychological systems in the execution of a task(s). Performance can be evaluated at the level of a biological system, psychological system, or at an integrated level for the whole person.

Phenotype—The observable characteristics of an organism including its physical properties, development and behavior, which result from the interaction of the genotype with the environment. Potential—a dimension of health—A person's capacities to grow, develop new capabilities, and mature in response to internal and external challenges, thereby producing new health states.

Typology of health—A hierarchical structure describing the processes of living systems that enable them to manage energy (energetics), monitor and interpret the environment (cognition), respond to internal and external environments (adaptation), reproduce (reproduction), and develop the capacities for modifying the social and physical environment (capabilities).
